# *Pneumocystis* and interactions with host immune receptors

**DOI:** 10.1371/journal.ppat.1006807

**Published:** 2018-02-22

**Authors:** Jennifer Claire Hoving

**Affiliations:** Institute of Infectious Disease and Molecular Medicine (IDM), Department of Pathology, Faculty of Health Sciences, University of Cape Town, Cape Town, South Africa; McGill University, CANADA

## Introduction

The majority of deaths from fungal infections occur in Africa. *Pneumocystis* is an unusual host-specific fungus that takes advantage of a weakened immune system and causes pneumonia in AIDS patients [1]. While *Pneumocystis* is thought to cause more than 200,000 AIDS-related deaths annually, it also contributes to more than 50,000 non–AIDS-related deaths [2]. Recent epidemiological data suggest that *Pneumocystis jirovecii* pneumonia (PCP) cases are increasing in both England and the United States due to the increased use of immune suppression therapy for cancer, transplant, and inflammatory disorder patients [3, 4]. PCP faces diagnostic and treatment challenges that are significantly exacerbated in resource-poor settings and compounded by the fact that the organism cannot be cultured in vitro. This review briefly summarizes key mechanisms of host recognition of *Pneumocystis* spp. and how these systems fail in an immunocompromised host.

## *Pneumocystis* spp. antigens recognized by the host

**Fig 1:**
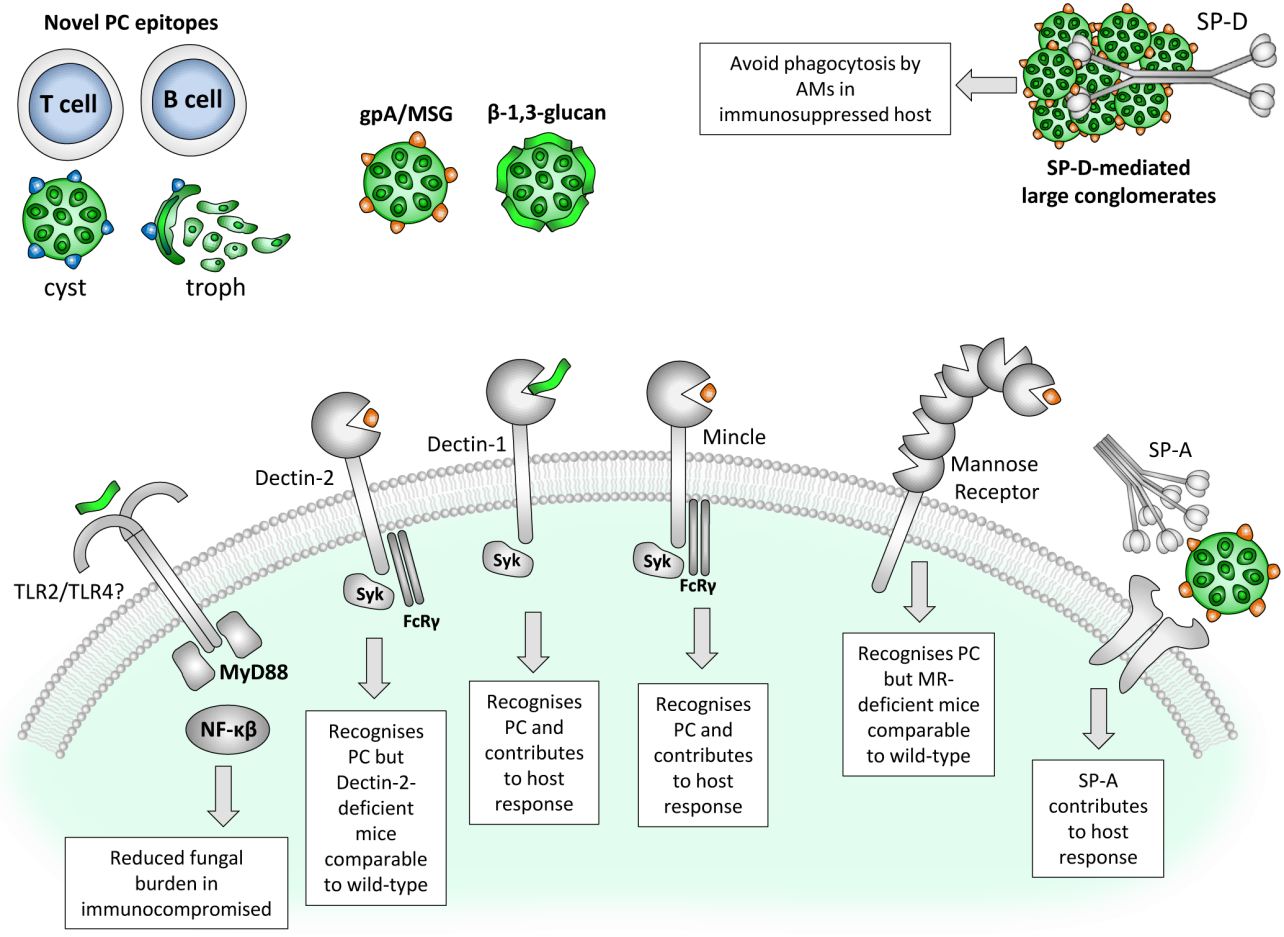
Host recognition of PC. MSG and β-1,3-glucan are the main surface proteins of PC recognized by the host; however, novel T-cell and B cell epitopes have been described. Soluble CLRs such as SP-A and SP-D influence PC clearance or escape, respectively. Mannose receptor and Dectin-2 have been shown to recognize PC but do not seem to contribute to clearance, shown in gene-deficient mice. In contrast, Dectin-1 and Mincle have been shown to recognize PC and, in immunocompromised mice, contribute to limiting disease progression. Similarly, MyD88 was shown to be involved in the host response in immunocompromised mice, potentially mediated through TLR2. AM, alveolar macrophage; CLR, C-type lectin receptor; FcRy, Fc receptor common gamma-chain; gpA, glycoprotein A; Mincle, Macrophage inducible Ca^2+^-dependent lectin receptor; MSG, major surface glycoprotein; MyD88, myeloid differentiation primary response 88; NF-κB, nuclear factor kappa-light-chain-enhancer of activated B cells; PC, *Pneumocystis*; SP, surfactant protein; Syk, Spleen tyrosine kinase; TLR, toll-like receptor [7,10,17,21,26,29,35].

There remain many unanswered questions surrounding the host recognition of *Pneumocystis* and the mechanisms of the immune response that result in clearing this obligate, opportunistic pathogen. However, combinations of both human and animal studies have provided key information in understanding some of the host receptors and pathways involved (Fig 1). First to be described, the major surface glycoprotein (MSG) glycoprotein A (gpA) is the most abundant *Pneumocystis* cell surface protein that interacts with host cells. Subsequently, β-1,3-glucan was identified and predominantly found in the thick wall of the ascus form. Furthermore, masking of β-1,3-glucan proteins was suggested to assist *Pneumocystis* in evading host recognition [5]. Kottom et al. have demonstrated that β-1,6-glucans are also present in the *P*. *carinii* cell wall and contribute to cellular activation during infection [6]. The identification of additional *Pneumocystis* antigens through innovative approaches, such as the surface proteomics analysis described by Zheng et al. used to identify novel T-cell and B cell epitopes, could provide new targets for *Pneumocystis*-specific therapy [7].

## Host recognition of *Pneumocystis* spp. by pattern recognition receptors

Pattern recognition receptors (PRRs) such as C-type lectin receptors (CLRs) and toll-like receptors (TLRs) have both been associated with fungal recognition. However, CLRs have been shown to play key roles in immunity to fungal pathogens. The mannose receptor (MR) was one of the first CLRs shown to recognize *Pneumocystis* [8] through binding with gpA [9] (Fig 1). While MR-deficient mice maintained their ability to clear *P*. *murina*, HIV-1 infection was shown to reduce binding and phagocytosis by macrophages associated with the down-regulation of MR [10,11]. Together, these data suggest that MR contributes to host recognition of *Pneumocystis*, which is compromised in HIV-1 infection. Surfactant protein (SP)-A and SP-D, collectins within the Group III C-type lectins, have also been linked with binding *Pneumocystis*. While SP-D binds to gpA and facilitates *P*. *carinii* attachment to macrophages, it does not seem to facilitate phagocytosis. Instead, SP-D appears to promote the aggregation of *Pneumocystis*, preventing phagocytosis and promoting infection [12]. For SP-A, in vitro studies provide conflicting results; however, studies using SP-A–deficient mice suggest that SP-A plays a protective role in vivo (Fig 1) [13]. For fungal infections, the best-characterized CLRs are the immunoreceptor tyrosine-based activation motif (ITAM)-coupled receptors Dectin-1, Dectin-2, and Macrophage inducible Ca^2+^-dependent lectin receptor (Mincle) [14,15]. Therefore, recent studies have scrutinized the role of these CLRs in initiating the host immune response to *Pneumocystis*.

## Dectin-1

A role for Dectin-1 in recognizing *Pneumocystis* was first highlighted by Steele et al. [16]. They found that Dectin-1 mediated phagocytosis and killing of *Pneumocystis* by macrophages and that blocking Dectin-1 inhibited binding and killing of *P*. *carinii*, while macrophages overexpressing Dectin-1 demonstrated increased binding to the organism. The role of Dectin-1–expressing macrophages in killing *Pneumocystis* seems most important in immunocompromised hosts, as shown by Saijo et al. [17]. Although Dectin-1–deficient mice were initially more sensitive to infection, shown by increased fungal burden within the first 2 weeks, they eventually cleared infection comparable to wild-type control mice. However, in Dectin-1–deficient mice immunosuppressed with cortisone acetate, *Pneumocystis* was able to persist at a higher rate than immunosuppressed wild-type mice, shown by an increase in the number of lung cysts. Therefore, the authors concluded that in the presence of an intact acquired immune system, Dectin-1 is not required for protection against chronic infection by *P*. *carinii* but that Dectin-1 is important in the absence of these responses. In a promising study involving Dectin-1, Ricks et al. generated Dectin-1 immunoadhesins that consisted of the carbohydrate-binding domain fused to the fragment crystallizable (Fc) regions of 4 subtypes of murine immunoglobulin G (IgG) [18]. They were able to reduce the ascus burden in the lung of immunocompromised nude mice, providing evidence for the potential of a Dectin-1–dependent treatment strategy for fungal infections.

## Mincle

The CLR Mincle recognizes pathogens and damaged cells and is known for its role in recognizing *Mycobacterium tuberculosis* (MTB) glycolipids, including mycobacterial cord factor trehalose-6,6’-dimycolate. Increasing evidence describes Mincle as an important fungal receptor for *Candida albicans* and *Malassezia* species [19, 20]. Recently, Kottom et al. have also described an important role for Mincle in the host response against *P*. *murina* [21]. Although, Mincle-deficient mice appear to be more sensitive to infection at the early stage, shown by increased fungal burdens, they maintain their ability to clear infection in mice with an intact immune response. However, CD4-depleted, Mincle-deficient mice were more susceptible to infection compared to their CD4-depleted wild-type counterparts. Therefore, this study highlights for the first time a significant role for Mincle in mediating host responses to *P*. *murina* in immunocompromised mice. Previous studies have highlighted the role Mincle plays in the signaling pathway of other PRRs. On the one hand, Mincle has been shown to contribute to the synergistic signaling with other CLRs. A potential breakthrough for the treatment of chromoblastomycosis, a lifelong fungal infection caused by *Foncesaea pedrosoi*, was described by Sousa Mda et al. [22]. Essentially, a defect in fungal recognition by Mincle could be restored by topical treatment with the TLR7 agonist Imiquimod in a mouse model. In a proof-of-principal study, the costimulation between TLR7 and Mincle resulted in fungal clearance in patients infected with *F*. *pedrosoi* [23]. On the other hand, fungi have been shown to exploit Mincle to suppress Dectin-1 and Dectin-2, redirecting CD4 T helper responses and thereby suppressing antifungal immunity [24, 25]. In response to *P*. *murina* infection, Mincle-deficient mice had increased expression of Dectin-1, Dectin-2, and C-Type Lectin Domain Family 4 Member D (CLEC4D, CLECSF8, or MCL), highlighting the role Mincle plays in down-regulating other CLRs in response to *Pneumocystis* [21].

## Other CLRs

Considering the increased expression of Dectin-1, Dectin-2, and CLEC4D (CLECSF8, MCL) in response to *Pneumocystis* infection, Kottom et al. recently also investigated the role of Dectin-2 in immunity to *Pneumocystis* [26]. They demonstrated that *Pneumocystis* does indeed bind Dectin-2 on macrophages and initiates the production of inflammatory cytokines such as interleukin 6 (IL-6) and tumor necrosis factor α (TNF-α). However, Dectin-2 seems not to play an essential role in controlling *Pneumocystis* infection in immunocompromised mice, as evidenced by Dectin-2–deficient, CD4-depleted mice having a similar outcome as wild-type control mice. Interestingly, in contrast to Mincle-deficient mice in which other CLR expression was increased, Dectin-2 deficiency resulted in significantly lower levels of Dectin-1 and Mincle expression. Another CLR to consider in *Pneumocystis* recognition is CLEC4D (CLECSF8, MCL). Considering that CLEC4D facilitates Mincle expression and signaling by forming a complex [27, 28] and that CLEC4D expression is also influenced by Dectin-2 [26], further studies here would provide valuable insight.

## TLRs

Conflicting evidence points to the involvement of TLRs in response to *Pneumocystis*. Myeloid differentiation primary response 88 (MyD88)-deficient mice with an otherwise intact immune system managed to control *P*. *murina* infection comparable to wild-type mice. This was demonstrated in both the bolus intratracheal inoculation model and a cohousing infection model [29, 30]. As seen with Dectin-1 and Mincle, immunosuppressing MyD88-deficient mice resulted in a more severe immunopathogenesis and higher fungal burden than wild-type mice. While these experiments highlight a potential role for TLRs in controlling *Pneumocystis*, the specific TLR remains to be determined. Previous papers have highlighted TLR2 (and potentially TLR4) but with conflicting data for this. Considering the overwhelming evidence supporting PRR cosignaling mechanisms and that Dectin-1 and TLR2 signal together in response to fungi [31,32], it is highly likely that the synergistic signaling of multiple PRRs rather than an individual receptor could be required for the host to clear *Pneumocystis*. Further studies investigating potential cosignaling mechanisms in response to *Pneumocystis* infection would be intriguing.

## Conclusion

Recent studies have provided key information as to the host recognition of and associated immune response to *Pneumocystis*, yet much remains unanswered. It has proven to be difficult to conclusively determine which host immune responses are involved in recognizing *Pneumocystis* and to distinguish the contribution of colonizing *Pneumocystis* organisms from those that drive pathology and disease. This is further confounded by the fact that the host immune response seems to be manipulated by *Pneumocystis*, as the trophic form is capable of dampening the inflammatory response by dendritic cells [33, 34]. However, evidence is clear that PRRs contribute to immune responses to *Pneumocystis* in the absence of an intact adaptive immune system. How these innate immune receptors shape the adaptive immune response remains elusive. While the role of CD4 cells in controlling *Pneumocystis* infection is well documented, it remains unclear which T helper cell subset is absolutely necessary for this control. Based on previous studies, it seems likely that there are redundant mechanisms involving multiple T helper cells and that the loss of one subclass is compensated for by the other subclasses. Further studies are required to fully understand the complexity of the host immune response to *Pneumocystis*.
